# Bad apples spoiling the metaphor? How and why self-serving leaders stir up counterproductive behaviors at work

**DOI:** 10.3389/fpsyg.2022.1008071

**Published:** 2023-01-26

**Authors:** Yajun Zhang, Shuai Peng, Jinsong Wang, Muhammad Naseer Akhtar, Yongqi Wang

**Affiliations:** ^1^School of Business Administration, Guizhou University of Finance and Economics, Guiyang, China; ^2^Royal Docks School of Business and Law, University of East London, London, United Kingdom; ^3^International College, Guangdong University of Foreign Studies, Guangzhou, China

**Keywords:** self-serving leadership, counterproductive work behavior, anger, traditionality, affective event theory

## Abstract

Self-serving leaders satisfy their self-interests at the cost of both employees and organizations, leading to declining organizational competitive advantage and performance. Drawing upon the affective events theory (AET), we constructed and examined a theoretical model of self-serving leadership influencing counterproductive work behavior (CWB), where traditionality plays a significant moderating role through the lens of anger as a mediator. Data were collected in three waves using a survey questionnaire distributed in three industries located in the Southwest district of China. Hierarchical regression analyses were conducted on a sample of 316 employees to test the hypothesized research model. The results showed that self-serving leadership triggers employee anger, which in turn causes CWB. Furthermore, traditionality plays a significant moderating role, in which employees with higher levels of traditionality feel less anger and show less CWB. Overall, research findings have clarified how and why self-serving leadership affects employees’ emotions (such as anger) and behavior (such as CWB), bringing new insights into the self-serving leadership and employee behavior literature. Research implications on the management of self-serving leadership, limitations, and future recommendations of research are also discussed.

## Introduction

Leadership effectiveness remains one of the most popular topics in organizational behavior research. Empowered by an organization, leadership is supposed to boost employee performance and assist in the long-term development of the organization (Bharanitharan et al., 2020). However, the prevalence of commercial scandals in recent years has demonstrated that organizational executives can act unethically (Decoster et al., 2021). Self-serving leaders advance their self-interests at the expense of employees and even the organization. Their actions include embezzling others’ resources, evading responsibilities, transferring illegal benefits, and abusing power (Rus et al., 2010a; Camps et al., 2012). Relevant reports reveal that when a firm is on the edge of bankruptcy, self-serving leaders seek every means to maximize their self-interests, such as enhancing their income, forcing salary cuts on staff, and transferring and embezzling corporate property (Rus et al., 2010a; Camps et al., 2012). Therefore, organizations must investigate the negative effects of self-serving leadership and how to effectively control it.

In recent years, scholars have focused on the negative effects of self-serving leadership. For instance, prior studies verified the relationship between self-serving leadership and employee turnover intentions (Decoster et al., 2014), deviant behavior (Decoster et al., 2021), team performance (Mao et al., 2019a), and team creativity (Peng et al., 2019), but not between self-serving leadership and employee CWB. It is exceedingly common for leaders in local organizations with tight hierarchies to utilize their authority to intrude on the resources of their subordinates (Schmid et al., 2019). Yet, unknown whether self-serving leadership will lead to employee CWB. Existing research on the mediating mechanism of self-serving leadership is mostly based on social information processing theory (Peng et al., 2019; Decoster et al., 2021), social exchange theory (Decoster et al., 2014; Mao et al., 2019b) and social learning theory (Peng et al., 2019). Other theoretical perspectives remain under-explored.

Leadership behavior such as abusive supervision has been proven to be the key antecedent of employee anger towards leaders (Mitchell et al., 2015; Simon et al., 2015), employee anger further triggers their own CWB (Rodell and Judge, 2009; Ilie et al., 2011; Liu et al., 2014; Zhou et al., 2018; Zhu and Zhang, 2021). Following the perspective of AET, prior research has not provided a clear answer to whether self-serving leaders indirectly influence CWB through employee anger. Regarding the boundary conditions of self-serving leadership, existing research mainly discusses perceived distributive fairness (Camps et al., 2012), task interdependence (Peng et al., 2019), ethical climate (Decoster et al., 2021), leader competence (Mao et al., 2019a), etc., but ignored the influence of the individual traits of employees. AET points out that emotional responses triggered by work events are significantly affected by individual traits (Weiss and Cropanzano, 1996). Similarly, traditionality has been found as the most important individual trait in eastern culture and varies substantially among people (Farh et al., 1997, 2007). Whether traditionality and self-serving leadership interact to affect employee anger needs further examination. To address these important gaps, we examine the influence of self-serving leadership on employee CWB through the lens of employee anger as a mediator and traditionality as a moderator in our hypothesized research model.

## Theoretical overview and hypotheses development

### Self-serving leadership and employee counterproductive work behavior

Self-serving leaders are leaders who appropriate the resources and interests of an organization and its employees to serve their self-interests (Decelles et al., 2012; Rus et al., 2012). Prior studies have found that leaders’ self-serving behavior not only weakens their managerial influence (Decoster et al., 2021), but also provokes negative emotions among employees (Camps et al., 2012), undermines their trust in leaders (Decoster et al., 2021), aggravates employee turnover intentions (Decoster et al., 2014), and even damages team psychological safety (Mao et al., 2019a; Peng et al., 2019). In addition, studies have found that with increasing managerial control, scope, and power, the self-serving behavior of leaders may become more rampant (Giurge et al., 2021). Due to the multiple destructive effects of leaders’ self-serving behavior, employees usually respond negatively to self-serving leaders (Decoster et al., 2021).

Employee CWB is defined as deliberate behavior that harms the interests of the organization (Dalal et al., 2009). CWB can be classified into two distinct categories: (1) Organizational counterproductive work behavior (CWBO): when the CWB is directed towards an organization, (2) Interpersonal counterproductive work behavior (CWBI): when the object of the CWB is an individual (Bai et al., 2016). The close link between leaders’ behavior and employee CWB has been supported by research findings (Hoobler and Brass, 2006). For example, negative leadership styles, such as abusive supervision (Wei and Si, 2013; Simon et al., 2015), can trigger employee CWB. Self-serving leaders prioritize personal interests and self-aspirations, and they frequently put self-interest first at work while ignoring employee needs and well-being (Rus et al., 2010a). When employees are treated unfairly, they tend to respond with bad actions to compensate for their lost interests (Jacobs et al., 2014). Thus, it is not surprising that when confronted with the self-serving behavior of leaders, employees show CWB such as wasting organizational resources, reducing input intensity, and venting to colleagues. Therefore, we propose the following hypothesis:

*H1a*: Self-serving leadership has a positive impact on employee CWBO.

*H1b*: Self-serving leadership has a positive impact on employee CWBI.

### The mediating role of employee anger

Anger is one of the most common negative emotions in the workplace, and it is typically caused by specific individuals such as leaders, colleagues, and subordinates (Chen and Spector, 1992). Anger, in general, is an emotional reaction to events that are repulsive or violate social rules (Carver and Harmon, 2009). In this study, we focus on employee anger toward leaders. Previous studies have shown that negative leadership behaviors such as abusive supervision are important causes of employee anger (Mitchell et al., 2015; Simon et al., 2015). Self-serving leaders often engage in selfish behaviors that harm others and benefit themselves and exasperate employees by seriously violating their interests (Decoster et al., 2014). Furthermore, leadership is regarded as the organization’s representative and the embodiment of its values and beliefs (Chen et al., 2019). The unethical behavior displayed by self-serving leaders will severely undermine the positive image and charm of leaders in the eyes of employees, which in turn cause employee anger (Camps et al., 2012). Therefore, self-serving leaders may positively predict employee anger.

When employee anger is provoked, they typically engage in aggressive or irrational behavior to vent or retaliate (Zhang et al., 2020). Zhou et al. (2018) also found that employee anger on the first day leads to CWB on the second. AET suggests workplace events indirectly affect individual cognition, attitude, and behavior (Weiss and Cropanzano, 1996). Previously, scholars have explored the mediating role of employee anger in the relationship between abusive supervision and CWB (Simon et al., 2015). Self-serving leadership, as an important negative event in the workplace, is bound to fuel employee anger and lead to employee CWB. In other words, employee anger mediates the relationship between self-serving leadership and employee CWB. Based on the above discussion, we suggest the following hypothesis:

*H2a*: Employee anger mediates the relationship between self-serving leadership and CWBO.

*H2b*: Employee anger mediates the relationship between self-serving leadership and CWBI.

### The moderating effect of traditionality

Traditionality is defined as an individual’s comprehensive and coordinated view of things, opinions, beliefs, and behavioral tendencies, the most common of which is obedience to authority, and conformity with norms and rules (Farh et al., 1997, 2007). Individuals differ greatly in their level of traditionality. Individuals with a high level of traditionality tend to “restrain themselves and follow the rule,” respect the hierarchy of traditional society, accept social norms and role obligations as their code of conduct, and unconditionally follow their superiors (Li et al., 2017). However, individuals with a low level of traditionality are the polar opposite (Farh et al., 1997, 2007). Traditionality has been shown to have a direct or indirect effect on several outcomes. For example, Tan et al. (2021) found that leadership traditionality was significantly negatively correlated with humorous behaviors.

Affective events theory points out that individual characteristics can have a moderating role in the process of workplace events affecting emotion (Weiss and Cropanzano, 1996). Employees with varying levels of traditionality may react differently to the same self-serving leader. First, high-traditionality employees obey authority due to their stronger sense of hierarchy and higher regard for precedence. They believe that a leader’s order cannot be disobeyed and feel a strong sense of responsibility to meet the leader’s demands and expectations unconditionally and uncritically (Farh et al., 2007). Employees with high traditionality do not dare to raise any objections even when the leader exhibits self-serving behavior. Second, highly-traditionality employees feature conservatism and endurance. They consider the complete and timely fulfillment of job duties as the role obligation that all subordinates should comply with, which is less dependent on external factors such as leadership situations (Farh et al., 1997, 2007). Consequently, the presence or absence of self-serving leadership behaviors has a limited effect on the affective responses of high-traditionality employees. Finally, high-traditionality employees are less sensitive to the relationships of social exchange. They are willing to maintain positive exchange relationships with their leaders regardless of how leaders treat them (Hui et al., 2004). As a result, they do not harbor much resentment even if the leader exploits them or even takes their credit (Chen and Aryee, 2007).

On the other hand, employees with low traditionality have a lower sense of hierarchy, hold liberal and egalitarian values, and follow the principle of equitable social exchange (Cheng et al., 2004; Farh et al., 2007). This makes them more sensitive to the self-serving behavior of leaders, and their anger levels vary more significantly with the rise and fall of self-serving behavior. High-traditionality employees accept differences in rank and status, closely adhere to role conventions, and trust and respect the leader’s authority (Chen and Aryee, 2007). Even if the leader acts for personal gain at work, they will not have much skepticism and dissatisfaction. In short, self-serving leadership has a smaller positive influence on the anger of employees with high traditionality (Farh et al., 1997, 2007). On the other hand, employees with low traditionality have a lower sense of hierarchy, refuse to accept their inferior status to leaders, and strive for ultimate fairness in workplace procedures, interactions, information, and distribution. They are sensitive to self-serving behaviors and exert strong emotional responses to them (Farh et al., 1997, 2007). In short, self-serving leadership has a greater positive impact on the anger of low-traditionality employees. Hence, we suggest the following hypothesis:

*H3*: The relationship between self-serving leadership and employee anger is moderated by traditionality. The higher the employee traditionality, the weaker the positive association between self-serving leadership and employee anger, and vice versa.

### Moderated mediation mechanism

When employee traditionality is low, self-serving leadership has a higher effect on his anger. At the same time, the indirect effect of self-serving leadership on his CWB through employee anger is greater too. When employee traditionality is high, the effect of self-serving leaders on their anger is weaker, and the indirect effect of self-serving leaders on their CWB through employee anger is also smaller. Therefore, we propose the following hypothesis:

*H4a*: Traditionality moderates the mediating effect of employee anger on self-serving leaders affecting CWBO. The higher the employee traditionality, the weaker the mediating effect; and vice versa.

*H4b*: Traditionality moderates the mediating effect of employee anger on self-serving leaders affecting CWBI. The higher the employee traditionality, the weaker the mediating effect; and vice versa.

**Figure 1:**
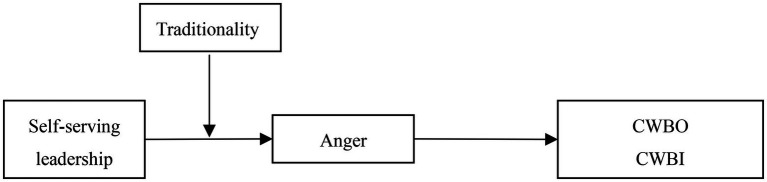
Research framework.

Based on the above discussion, the research framework constructed in this study is shown in Figure 1.

## Materials and methods

### Research design and procedure

Data were collected from paper questionnaires filled out by employees of several organizations (including retail, finance, and IT) located in the southwest district of China. The paper-based survey questionnaires were distributed at three points in time, with a 2-week interval between each data point collection. In order to ensure the quality of the questionnaires, respondents were required to have worked with their immediate supervisors for at least 3 months. Before distributing the questionnaire, the researchers informed all participants that the content of the questionnaire would only be used for academic research and will be kept completely confidential and anonymous. The researchers sent three small envelopes and one large envelope with questionnaires and telephone codes to the employees and asked them to fill in the survey according to the prescribed three-time points.

At T1, participants were asked to fill out demographics, self-serving leadership, and traditionality. 400 questionnaires were handed out and 372 were returned. At T2, we asked those who have completed the questionnaire at T1 to rate anger. We sent out 372 copies and 356 were collected. Similarly, at T3, we asked those who have completed the survey at T2 to report on CWBO as well as CWBI. Out of 356 questionnaires distributed, 339 questionnaires were returned. Upon competition, the questionnaires were returned to researchers in a large envelope. After excluding responses with random answers, missing data, and unmatched time points, a total of 316 questionnaires was the final useable sample for further analysis. The respondents were mainly male (189 men, accounting for 59.8% of the total sample). The age ranged from 26 to 35 for the majority (71.5%) and amount to 226 people. 169 respondents (53.5%) completed undergraduate education, and 103 participants (32.6%) have worked with their direct leader for 7–12 months.

### Measures

All the variables in our hypothesized research model are based on five-point Likert-type scales validated and developed by organizational scholars.

Self-serving leadership was assessed by adopting a 4-items scale developed by Camps et al. (2012). The sample item was, “My supervisor uses resources of the company for him/herself.” The Cronbach’s alpha was 0.93.

Anger was measured by adopting the scale devised by Chen and Spector (1992) covering 4 items, such as “*The selfish behavior of the supervisor irritates me*.” The Cronbach’s alpha for anger was 0.95.

Traditionality was evaluated with the scale designed by Farh et al. (2007), based on 5 items. The sample item was, “Following the instructions of a senior person is the best way to avoid mistakes “. The Cronbach’s alpha score for traditionality was 0.90.

Counterproductive work behavior was measured with the scale proposed by Dalal et al. (2009) based on 12 items. Half of these items were CWBO, such as “*I took an unnecessary break at work*.” The Cronbach’s alpha score was 0.93. Another half was items of CWBI, such as “*I tried to harm my supervisor/a coworker because of work*.” The Cronbach’s alpha value for this scale was 0.93.

Based on the research of Mao et al. (2019b), we controlled for employees’ gender, age, education level, and tenure with a leader.

## Results

### Confirmatory factor analysis

We used AMOS v.26 to conduct confirmatory factor analysis to examine the discriminant validity of self-serving leadership, anger, traditionality, CWBO, and CWBI. As shown in Table 1, the five-factor model showed the best fit and is significantly better than other comparable models (*χ*^2^/*df* = 2.30, CFI = 0.95, TLI = 0.95, IFI = 0.95, RMSEA = 0.06). Therefore, the five latent variables examined in this paper have good discriminant validity.

**Table 1:** Results of confirmatory factor analysis.

	*χ^2^*	*df*	*χ^2^/df*	*∆χ^2^*(*∆df*)	CFI	TLI	IFI	RMSEA
Five-factor model	610.12	265	2.30		0.95	0.95	0.95	0.06
Four-factor model	916.15	269	3.41	306.03^***^ (4)	0.91	0.90	0.91	0.09
Three-factor model	1786.07	272	6.57	1175.95^***^ (7)	0.79	0.77	0.79	0.13
Two-factor model	2795.59	274	10.20	2185.47^***^ (9)	0.65	0.61	0.65	0.17
One-factor model	3784.57	275	13.76	3174.45^***^ (10)	0.51	0.47	0.51	0.20
Zero-factor model	7455.54	300	24.85					

*n* = 316; ^***^p < 0.001. Five-factor model: self-serving leadership, anger, traditionality, CWBO, CWBI; Four-factor model: self-serving leadership, anger, traditionality, CWBO+ CWBI; Three-factor model: self-serving leadership + anger, traditionality, CWBO + CWBI; Two-factor model: self-serving leadership + anger + traditionality, CWBO + CWBI; One-factor model: Self-serving leadership + anger + traditionality + CWBO + CWBI. “+” combing the factors.

### Descriptive statistical analysis

We utilized SPSS v.26 to conduct descriptive statistical analysis. The mean, standard deviation, and correlation coefficient of variables including self-serving leadership, anger, traditionality, CWBO, and CWBI are shown in Table 2.

**Table 2:** Results of descriptive statistical analysis.

	1	2	3	4	5	6	7	8	9
1. Sex									
2. Age	0.10								
3. Education level	−0.11^*^	0.06							
4. Tenure with leader	−0.02	0.25^**^	0.03						
5. Self-serving leadership	0.11	−0.05	0.08	0.06					
6. Anger	−0.07	−0.02	−0.00	0.00	0.46^**^				
7. Traditionality	0.14^*^	0.01	−0.01	−0.15^**^	0.21^**^	0.14^*^			
8. *CWBO*	0.10	−0.15^**^	−0.07	−0.09	0.40^**^	0.54^**^	0.11^*^		
9. *CWBI*	0.16^**^	−0.12^*^	−0.06	−0.07	0.45^**^	0.52^**^	0.16^**^	0.77^**^	
Mean	0.40	2.13	2.32	2.21	1.90	2.07	2.29	1.83	1.56
SD	0.49	0.62	0.60	1.04	1.07	1.08	1.01	0.90	0.85

*n* = 316; ^*^p < 0.05, ^**^p < 0.01. Sex: Male (0), female (1); Age: ≤25 years (1), 26–35 years (2), 36–45 years (3), ≥46 years (4); Education level: Junior college (1), undergraduate degree (2), postgraduate degree (3); Tenure with leader: ≤6 months (1), 7–12 months (2), 13–24 months (3), ≥25 months (4).

### Hypotheses testing

To test the hypotheses, we used SPSS v.26 to conduct a hierarchical regression analysis (the results are shown in Table 3). We hypothesized that self-serving leadership will be positively related to CWBO. As presented in Model 4, self-serving leadership has a significant positive impact on employee CWBO (β *=* 0.40, *p <* 0.001). Thus, H1a was supported. In H1b, it was hypothesized that self-serving leadership will be positively related to CWBI. As shown in Model 7, self-serving leadership has a significant positive impact on employee CWBI (β *=* 0.44, *p* < 0.001), which supported H1b.

**Table 3:** Results of hierarchical regression analysis.

Variables	Anger		*CWBO*			*CWBI*		
	Model 1	Model 2	Model 3	Model 4	Model 5	Model 6	Model 7	Model 8
Sex	−0.13^*^	−0.11^*^	0.10	0.05	0.11^*^	0.16^**^	0.11^*^	0.16^**^
Age	0.03	0.04	−0.14^*^	−0.11^*^	−0.12^*^	−0.12^*^	−0.08	−0.09^*^
Education level	−0.06	−0.06	−0.05	−0.09	−0.06	−0.04	−0.08	−0.06
Tenure with leader	−0.03	−0.04	−0.06	−0.09	−0.07	−0.04	−0.07	−0.06
Self-serving leadership	0.48^***^	0.53^***^		0.40^***^	0.17^**^		0.44^***^	0.24^***^
Anger					0.47^***^			0.42^***^
Traditionality		0.07						
Self-serving leadership ×Traditionality		−0.21^***^						
*R* ^2^	0.23	0.27	0.04	0.19	0.36	0.05	0.23	0.37
*ΔR* ^2^	–	0.04	–	0.15	0.17	–	0.19	0.14
*F*	18.35^***^	8.26^***^	3.158^*^	58.21^***^	80.94^***^	3.635^**^	75.94^***^	67.75^***^

*n* = 316; ^*^p < 0.05, ^**^p < 0.01, ^***^p < 0.001.

We argued that anger mediates the relationship between self-serving leadership and CWBO. As shown in Model 5, employee anger positively affects CWBO (β = 0.47, *p* < 0.001), but the positive effect of self-serving leadership on employee CWBO was significantly weakened (β = 0.17, *p* < 0.001). This suggests that employee anger mediates the relationship between self-serving leadership and CWBO. Thus, H2a was initially supported. Next, we examined the mediating effect of employee anger between self-serving leadership and CWBI. According to Model 8, employee anger positively affects CWBI (β = 0.42, *p* < 0.001), but the positive effect of self-serving leadership on employee CWBI was significantly weakened (β = 0.24, *p* < 0.001). This suggests that employee anger mediates the relationship between self-serving leadership and CWBI. Thus, H2b was also supported.

The Bootstrap method as recommended by Preacher and Selig (2012) was used to further test the significance of the mediating effect of employee anger on the relationship between self-serving leadership and CWBO and CWBI. The results showed that the mediating effects of self-serving leadership affecting CWBO and CWBI through employee anger were 0.20 and 0.17 respectively, and the 99% confidence interval (CI) is [0.12, 0.31] and [0.09, 0.27], excluding ‘0’ in between. Hence, it was confirmed that the mediating effect of employee anger was significant, i.e., H2a and H2b were fully supported.

### Analysis of moderating effects

**Figure 2:**
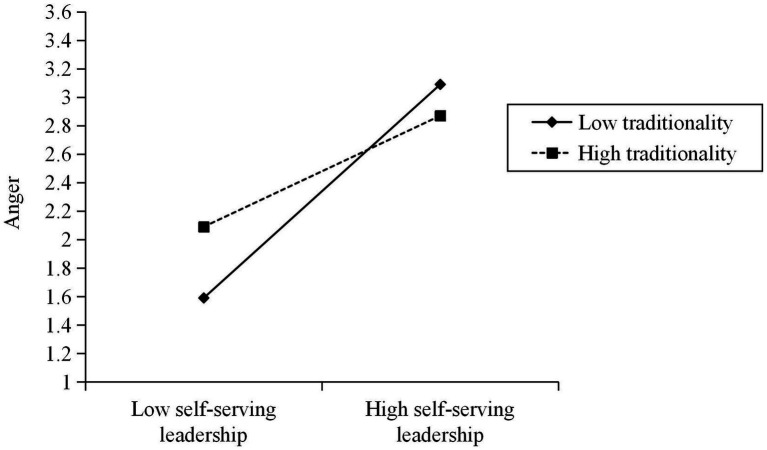
The moderating effect of traditionality on the relationship between self-serving leadership and anger.

To test the moderating effect of traditionality, gender, age, education level, working time with a leader, self-serving leadership, traditionality, and interaction terms were simultaneously entered into the regression equation with employee anger as the dependent variable. According to Model 2, the interaction between self-serving leadership and traditionality has a significant negative impact on employee anger (β = −0.21, *p*/ < 0.001), which indicates that traditionality negatively moderates the relationship between self-serving leadership and employee anger. Following the suggestion of Aiken and West (1991), the researchers created a moderating effect figure (see Figure 2). The results of simple slope analysis show that: the lower the employee traditionality, the stronger the positive effect of self-serving leadership on his anger (β = 0.75, *p* < 0.001); and the higher the employee traditionality, the weaker the positive effect of self-serving leadership on his anger (β = 0.39, *p* < 0.001). H3 has received preliminary support.

The moderated mediation effect was tested using the bootstrap method (Preacher and Selig, 2012), and the results are shown in Table 4. As presented in Table 4, the positive association between self-serving leadership and employee anger was significant at both high (*b* = 0.40, 95% CI = [0.27, 0.55]) and low (*b* = 0.75, 95% CI = [0.58, 0.92]) traditionality levels. At different traditionality levels, the variance in the influence of self-serving leadership on employee anger was also significant (*b* = −0.35, 95% CI = [−0.52, −0.11]), offering complete support for H3. In addition, when the outcome variable was CWBO, the mediating effect of employee anger was significant at both high (*b* = 0.16, 95% CI = [0.10, 0.24]) and low (*b* = 0.30, 95% CI = [0.21, 0.42]) level of traditionality. At different levels of employee tradition, the difference in the mediating effect of employee anger between self-serving leadership and CWBO was also significant (*b* = −0.14, 95% CI = [−0.23, −0.06]). These results showed full support for H4a. When the outcome variable was CWBI, the mediating effect of employee anger was significant at both high (*b* = 0.14, 95% CI = [0.08, 0.21]) and low (*b* = 0.26, 95% CI = [0.17, 0.37]) traditionality level. At different levels of employee traditionality, the mediating effect of employee anger between self-serving leadership and CWBI was also significant (*b* = −0.12, 95% CI = [− 0.20, −0.05]). Thus, H4b was supported.

**Table 4:** Results of moderated mediation analysis.

Dependent variables	Moderator	Self-serving leadership → Anger	Indirect effect	Direct effect	Total effect
*CWBO*	High traditionality	0.40^*^ [0.27, 0.55]	0.16^*^ [0.10, 0.24]	0.18^*^ [0.04, 0.32]	0.34^*^ [0.21, 0.47]
	Low traditionality	0.75^*^ [0.58, 0.92]	0.30^*^ [0.21, 0.42]	0.08 [−0.06, 0.23]	0.39^*^ [0.24, 0.55]
	Differences (*Δ*)	−0.35^*^ [−0.52, −0.11]	−0.14^*^ [−0.23, −0.06]	0.10 [−0.10, 0.28]	−0.05 [−0.25, 0.15]
*CWBI*	High traditionality	0.40^*^ [0.27, 0.55]	0.14^*^ [0.08, 0.21]	0.23^*^ [0.09, 0.38]	0.36^*^ [0.23, 0.53]
	Low traditionality	0.75^*^ [0.58, 0.92]	0.26^*^ [0.17, 0.37]	0.10 [−0.04, 0.27]	0.36^*^ [0.20, 0.53]
	Differences (*Δ*)	−0.35^*^ [−0.52, −0.11]	−0.12^*^ [−0.20, −0.05]	0.13 [−0.06, 0.34]	0.01 [−0.20, 0.24]

*n* = 316; ^*^p < 0.05, 95% confidence interval in parentheses.

## Discussion

The main findings of this research are: (1) self-serving leadership has a significant positive impact on employee CWB; (2) self-serving leadership indirectly affects CWB through employee anger; (3) the influence of self-serving leadership on employee anger is negatively moderated by traditionality, which means the lower the traditionality of employee, the stronger the positive effect of self-serving leadership on employee anger, and vice versa; (4) traditionality negatively moderates the indirect effect of self-serving leadership on CWB through employee anger, which means the lower the employee traditionality, the stronger the indirect effect, and vice versa.

### Theoretical contribution

First, we found that self-serving leadership exerts a significant positive impact on employee CWB (i.e., both CWBO and CWBI). A systematic review of the literature finds that the focus of early scholars’ research on self-serving leadership was to explore its antecedents. These studies confirmed that power (Rus et al., 2012), leadership self-concept (Wisse and Rus, 2012), leadership self-definition (Rus et al., 2010b), and leader psychopathy (Barelds et al., 2018) are important antecedents of self-serving leadership. However, research on the effects of self-serving leadership is relatively scarce. For example, Mao et al. (2019a) and Peng et al. (2019) found that self-serving leadership is a significant negative predictor of team performance and team creativity. Decoster et al. (2021) found that self-serving leaders aggravate the deviant behavior of employees. We attempted to examine self-serving leadership and employee CWB and found that there is a significant positive relationship between the two. Our study expands the research on the outcome variables of self-serving leaders and the antecedent variables of employee CWB. In addition, this study responds to the call of Peng et al. (2019) for further inquiries into the varied influence mechanisms of self-serving leaders at different levels.

Second, we discovered that self-serving leaders provoke CWB through employee anger. An in-depth analysis of the literature on the mediating mechanism of self-serving leadership affecting subordinates’ behavior showed that scholars have mainly discussed the issue from two theoretical perspectives; the first is the theory of social information processing and relevant mediating variables, e.g., team psychological safety (Mao et al., 2019a; Peng et al., 2019), and the second is social exchange theory as well as relevant mediating variables, e.g., affective commitment (Decoster et al., 2014; Mao et al., 2019b) and trust in leadership (Decoster et al., 2021). Based on AET, our study not only opens the ‘black box’ of how self-serving leadership affects employee CWB from a new theoretical perspective but also expands the application of AET by examining the mediating role of anger.

Finally, our study explains the boundary condition under which self-serving leadership affects employee CWB. A detailed review of the literature on the moderators of self-serving leadership showed that from the perspective of fairness, some scholars have explored the moderating role of perceived distributive justice (Camps et al., 2012). Similarly, from the perspective of traits of organization, leadership, and team, other researchers explored the moderating role of ethical climate (Decoster et al., 2021), leader competence (Mao et al., 2019a), and task interdependence (Peng et al., 2019); and from the perspective of cultural values, the moderating effect of power distance orientation (Mao et al., 2019b) was explored. However, employee traits did not receive due attention. We found that traditionality negatively moderates both the relationship between self-serving leadership and employee anger, and the indirect effect of self-serving leadership on employee CWB through his anger. In addition, our study broadens the research on traditionality as a boundary condition.

### Practical implications

First, this study confirmed that self-serving leadership induces employee CWB, suggesting that measures need to be taken to suppress the reoccurrence of such leadership behavior. Leaders should be the paragons of organizational ethics, not demonstrators of unethical behavior. To avoid the negative impact of self-serving leadership on employees, other than evaluating mental health and business ability in the recruitment of managerial positions, organizations should conduct leadership style tests to screen out candidates with self-serving behavior. For leaders who are already on the post, in addition to restraining their behavior by rules and regulations, training should also be in place to reshape their behavior. Second, we also found that employee anger mediates the relationship between self-serving leadership and employee CWB, suggesting that organizations can avoid the indirect influence of self-serving leadership on CWB by taking measures to reduce employee anger. These measures begin with arranging other colleagues to appease employees in anger.

Professional staff can be hired for emotional counseling when colleagues failed to help. An anger room can be set up for employees to vent their emotions by smashing objects like porcelain bowls and wine bottles. Training and exercise on emotional management skills can also be organized to increase employees’ tolerance for negative work events. Third, our study confirms that compared with high-traditionality employees, self-serving leadership has a stronger positive impact on the anger of low-traditionality employees, which suggests that organizations should pay attention to individual differences and treat employees of different traditionality levels differently. For example, questionnaires can be used to evaluate the traditionality level of prospective recruits. During team building, high-traditionality employees can be matched with highly self-serving leaders, and low self-interest leaders can be arranged to supervise low-traditionality employees.

### Research limitations and future recommendations

First of all, this study examined the mediating role of anger between self-serving leadership and CWB only from the perspective of AET, ignoring other potential mediating mechanisms. Self-serving leadership can cause a certain degree of resource depletion among subordinates (Mao et al., 2019a). In order to replenish lost resources, employees are likely to engage in CWB (Penney et al., 2011). Therefore, future studies can draw on the conservation of resources theory to analyze and investigate whether resource depletion mediates the relation between self-serving leadership and CWB.

Second, this study focused on the moderating role of traditionality in the first stage but ignored the boundary conditions between anger and CWB. Previous studies have found that conscientiousness, extraversion, and agreeableness are important predictors of CWB (Schmidt et al., 2011; Oh et al., 2014). Therefore, the big-five theory of personality could guide further investigation into the moderating effects of personality traits such as conscientiousness on the relationship between anger and CWB.

Third, CWB is by nature concealed and difficult to be observed by others (Dalal et al., 2009). Therefore, all the variables in this study were reported by employees, which may lead to the limitations of common method bias and social desirability. Therefore, further studies could invite leaders and colleagues who have worked with the employees for some time to evaluate their level of CWB.

## Conclusion

Drawing upon the affective event theory, we discovered that self-serving leadership induces CWB (including CWBO and CWBI). The consequences of this relationship are contingent upon employee anger and varying levels of traditionality. Our study examined the unexplored relationships between self-serving leadership and CWB through the mediating mechanism of employee anger and the varying levels of traditionality and offers new insights and future directions for self-serving leadership and CWB.

## Data availability statement

The raw data supporting the conclusions of this article will be made available by the authors, without undue reservation.

## Ethics statement

Ethical approval was not provided for this study on human participants because an ethics approval was not required as per our institution’s guidelines and national regulations. The patients/ participants provided their written informed consent to participate in this study.

## Author contributions

YZ: conceptualization, data curation, and writing-original draft. SP: writing-original draft. JW: data analysis, editing. MA: data analysis, writing, and editing. YW: writing and editing. All authors contributed to the article and approved the submitted version.

## Funding

We acknowledge the financial support from the National Social Science Foundation of China (No. 19BGL112).

## Conflict of interest

The authors declare that the research was conducted in the absence of any commercial or financial relationships that could be construed as a potential conflict of interest.

## Publisher’s note

All claims expressed in this article are solely those of the authors and do not necessarily represent those of their affiliated organizations, or those of the publisher, the editors and the reviewers. Any product that may be evaluated in this article, or claim that may be made by its manufacturer, is not guaranteed or endorsed by the publisher.
